# A kinase inhibitor library screen identifies novel enzymes involved in ototoxic damage to the murine organ of Corti

**DOI:** 10.1371/journal.pone.0186001

**Published:** 2017-10-19

**Authors:** Matthew Ryals, Kwang Pak, Rahul Jalota, Arwa Kurabi, Allen F. Ryan

**Affiliations:** 1 Department of Surgery/Otolaryngology, University of California, San Diego, School of Medicine, La Jolla, California, United States of America; 2 Research Service, Veterans Administration Medical Center, San Diego, California, United States of America; Harvard University, UNITED STATES

## Abstract

Ototoxicity is a significant side effect of a number of drugs, including the aminoglycoside antibiotics and platinum-based chemotherapeutic agents that are used to treat life-threatening illnesses. Although much progress has been made, the mechanisms that lead to ototoxic loss of inner ear sensory hair cells (HCs) remains incompletely understood. Given the critical role of protein phosphorylation in intracellular processes, including both damage and survival signaling, we screened a library of kinase inhibitors targeting members of all the major families in the kinome. Micro-explants from the organ of Corti of mice in which only the sensory cells express GFP were exposed to 200 μM of the ototoxic aminoglycoside gentamicin with or without three dosages of each kinase inhibitor. The loss of sensory cells was compared to that seen with gentamicin alone, or without treatment. Of the 160 inhibitors, 15 exhibited a statistically significant protective effect, while 3 significantly enhanced HC loss. The results confirm some previous studies of kinase involvement in HC damage and survival, and also highlight several novel potential kinase pathway contributions to ototoxicity.

## Introduction

Patients who receive ototoxic drugs, including aminoglycosides and platinum-based anticancer agents, frequently experience permanent sensorineural hearing loss. Ototoxicity due to aminoglycoside treatment for multi-drug resistant tuberculosis can exceed 50% [1–2], and children treated for cystic fibrosis nearly 25% [3] while the incidence of hearing loss following cisplatin or carboplatin treatment exceed 60% [4–5]. The primary targets of this ototoxicity are the sensory cells of the inner ear, known as hair cells (HCs), with the outer HCs being more sensitive than the inner HCs (e.g. [6]).

Understanding the cellular mechanisms that underlie ototoxic damage to HCs remains an area of active investigation. The generation of reactive oxygen species (ROS) originating from the mitochondria of HCs have been strongly linked to the early stages of ototoxicity (e.g. [7]). Downstream, the activation of the pro-apoptotic kRas/cdc42/JNK signaling cascade leading to the phosphorylation of cJun has also been implicated (e.g. [8]), as have apoptosis (e.g. [9]) and necroptosis [10]. There are numerous studies showing a protective effect of various pharmacological agents directed against these cellular processes (e.g. [8, 11–13]). However, given the complexity of intracellular signaling and other events, it seems likely that additional processes contribute to ototoxic HC damage.

To discover such processes, several systems have been developed to screen for otoprotective agents. These include, in particular, immortalized mammalian cell lines derived from inner ear cells (e.g. [14]) and the zebrafish lateral line (e.g. [15]). These systems have been highly useful and have contributed significantly to our knowledge of HC loss mechanisms. However, they do not directly address the highly specialized mammalian HCs. Moreover, given that the unique mammalian outer HC is the most sensitive HC to a variety of forms of damage (e.g. [6,16,17]), we felt that a screen that included this cell type would be useful in illuminating mechanisms of mammalian HC loss.

The purpose of the present study was to develop an assay using the mammalian organ of Corti (oC), which could evaluate a variety of compounds to test for potential modification of the HC toxicity of gentamicin, a powerfully ototoxic aminoglycoside antibiotic. A strong dosage was chosen that produced total or near-total HC loss. The loss occurred over a time course of several days as opposed to hours, to more closely mimic the time course of HC loss during *in vivo* ototoxicity. We chose to evaluate the assay using a library of kinase inhibitors. Phosphorylation is an important means of post-translational modification of proteins, which plays a major role in intracellular signaling and other cellular processes (e.g. [18,19]). It therefore seemed possible that a screen of inhibitors targeting all the major families of the mammalian kinome would identify novel processes involved in ototoxic HC damage. In addition, if successfully developed, the assay could also be useful for screening the effects of other pharmacological agents on mammalian HC damage.

## Materials and methods

### Animals

Experiments were performed on transgenic mice, in which eGFP (enhanced green fluorescent protein) was selectively expressed in HCs under the control of a *pou4f3* promoter construct [20], bred onto a CBA background. The transgenic mice were generated in our laboratory and bred for use. All experiments were performed to National Institutes of Health guidelines and approved by the Institutional Animal Care and Use Committee of the VA San Diego Medical Center. Animals were held in standard rodent boxes in containing two females and one male. Upon evidence of pregnancy, the male was removed. Rodent chow and water were freely available, and environmental enrichment was provided. Mouse pups were deeply anesthetized with rodent cocktail (2.0 mg/kg xylazine and 40.0 mg/kg ketamine i.m.) prior to sacrifice to dissect the organ of Corti.

### Kinase inhibitor libraries

The kinase inhibitory compounds used (EMD Calbiochem Kinase Inhibitor Libraries) consisted of two libraries (Libraries I and II) of 80 inhibitory compounds each. The libraries were provided in two separate sets of plates, and all compounds were provided in DMSO. Each library included both DMSO and blank wells for controls.

### *In vitro* screening

**Fig 1:**
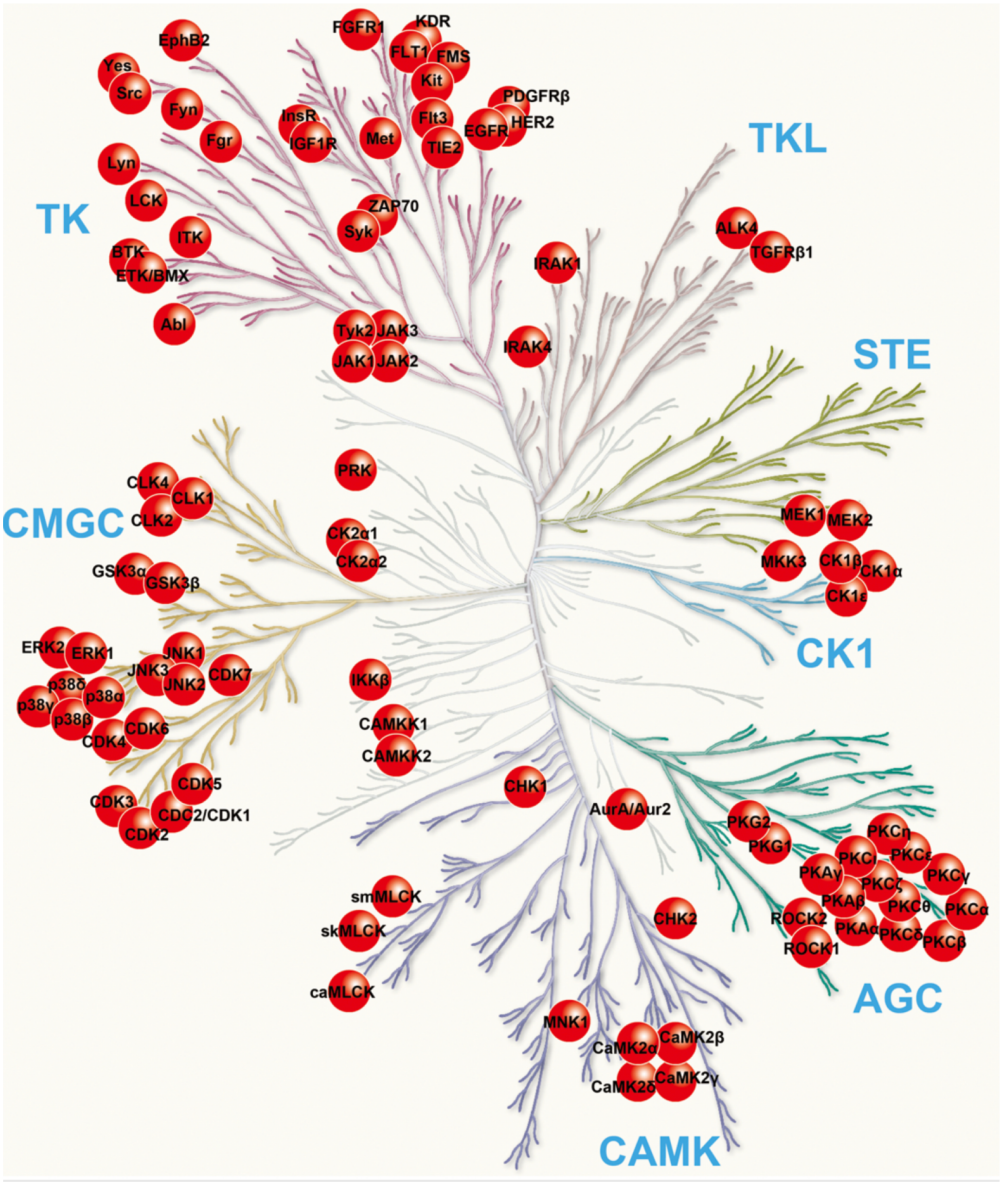
Distribution of tested inhibitors across the kinome. The inhibitor targets are indicated in red. The kinome diagram is adapted, with permission, from Cell Signaling Technologies.

The oC was dissected from the cochleas of 3–5 day old *pou4f3*/eGFP mouse pups. The apical region of each epithelium, which is relatively insensitive to aminoglycoside toxicity, was discarded. The basal and middle regions of the epithelium were cut into micro-explants, consisting of approximately 20 inner HCs and the associated outer HCs, using a diamond scalpel. Preliminary testing established that HCs in basal and middle turn micro-explants behaved similarly in response to 200 μM gentamicin. The micro-explants were plated on in flat-bottom, 96-well plates in media consisting of DMEM/F-12, penicillin and 5% FBS. The cultures were pretreated, beginning at the initiation of culture, for 24 hrs with one of the 160 kinase inhibitors at concentrations of 50, 100, or 500 nM. The distributions of the library kinase inhibitor targets within the major classes of the kinome are illustrated graphically in Fig 1. In addition, the inhibitors are listed in S1 Table. Each inhibitor was diluted in media with DMSO, with the total amount of DMSO in the culture media adjusted to 0.1% in each well. Media containing 200 μM gentamicin and one inhibitor concentration plus DMSO was substituted after the overnight pretreatment, on day 0 (D0), and the micro-explants were then cultured for 72 hrs through D3. Negative controls were cultured from D0-D3 in media alone with no inhibitors, while positive controls were cultured in media with the addition of 200 μM gentamicin with no inhibitors beginning on D0. Both controls contained 0.1% DMSO. Each inhibitor concentration and control condition was performed in triplicate, thus each 96-well plate tested 7 inhibitors plus controls. The limited size of the testing plates required verification that the control conditions were consistent from plate to plate, and thus both controls were included in each plate. GFP-positive cells were imaged by inverted fluorescence microcopy on each day of treatment.

### Validation and analysis

Inhibitor hits were identified qualitatively during the first round of inhibitor screening, based on visually obvious increases in GFP-positive HC survival. Following this initial identification of compounds of interest, repeat plates were prepared in an identical manner for all hits. Two additional concentrations of each compound, 10 nM and 1000 nM, were also tested. Finally, micro-explants showing either HC protection or enhanced HC damage were treated with the compound alone at 1000 nM to evaluate toxicity. HC counts, including both inner and outer HCs, were then performed for both the first and repeat tests. Survival curves were generated for each condition, by normalizing HC counts to the number of HCs present on D0 (at the initiation of gentamicin treatment), since each micro-explant contained a slightly different starting HC number. Any micro-explants that did not attach and flatten in the well by D0 were excluded, as HC counts could not be accurately quantified at this time. Statistical analysis of HC counts was performed using GraphPad Prism 6, StatView 5. Inhibitor treatment and control group data at each time point were analyzed by one-way ANOVA with posthoc Fisher’s least significant difference and Tukey tests (with Bonferroni correction for multiple comparisons) to identify significant differences between treatment and control groups.

## Results

### Control micro-explants

**Fig 2:**
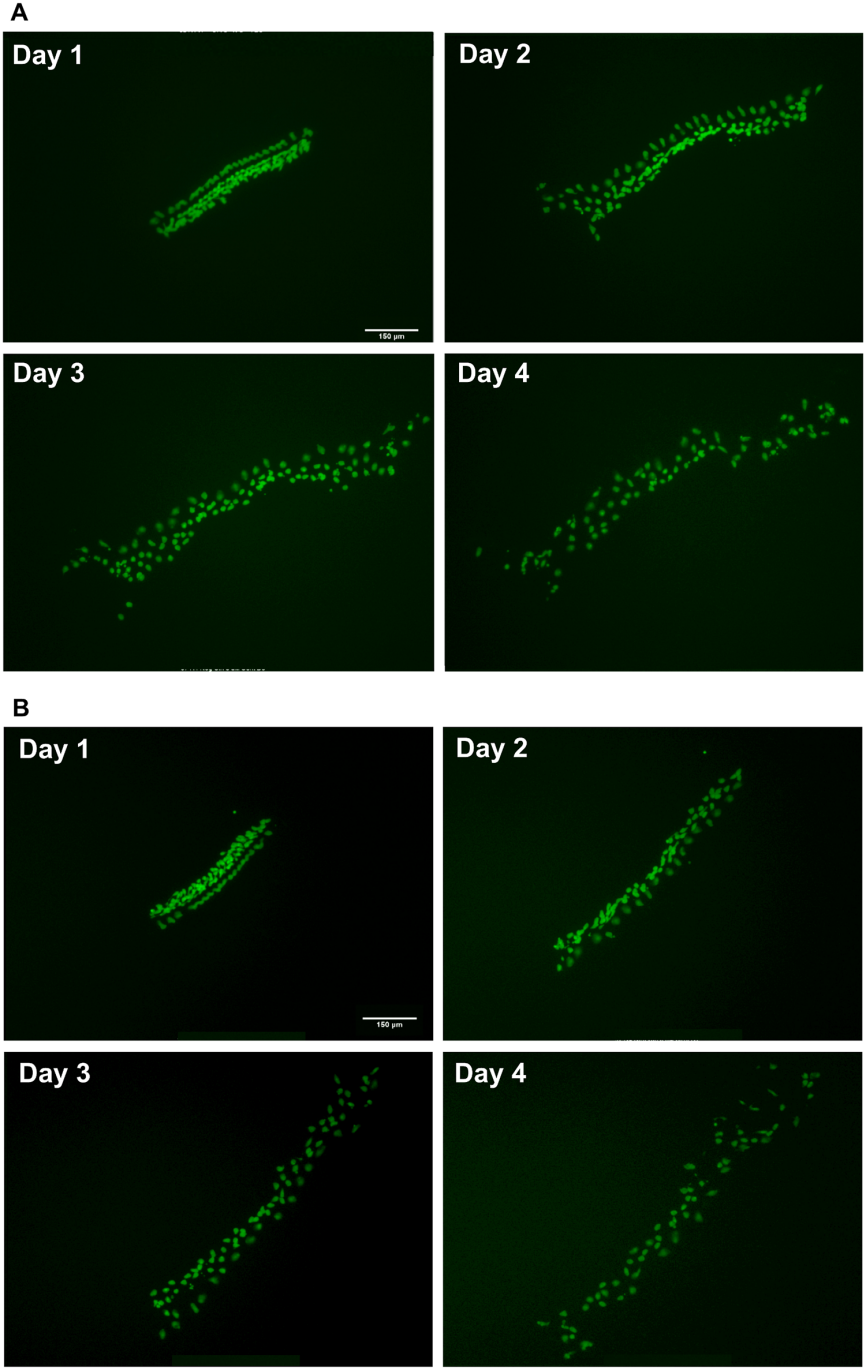
Negative control cultures. Montages of representative negative control (untreated) oC micro-explants from the screen of Library I (**2A**) and Library II (**2B**), from D1 through D4. HC loss was minimal through D3, with some loss by D4, for both libraries. (Fluorescence intensity for this and the following figures has been slightly enhanced for later days, as HC GFP intensity faded over time.)

**Fig 3:**
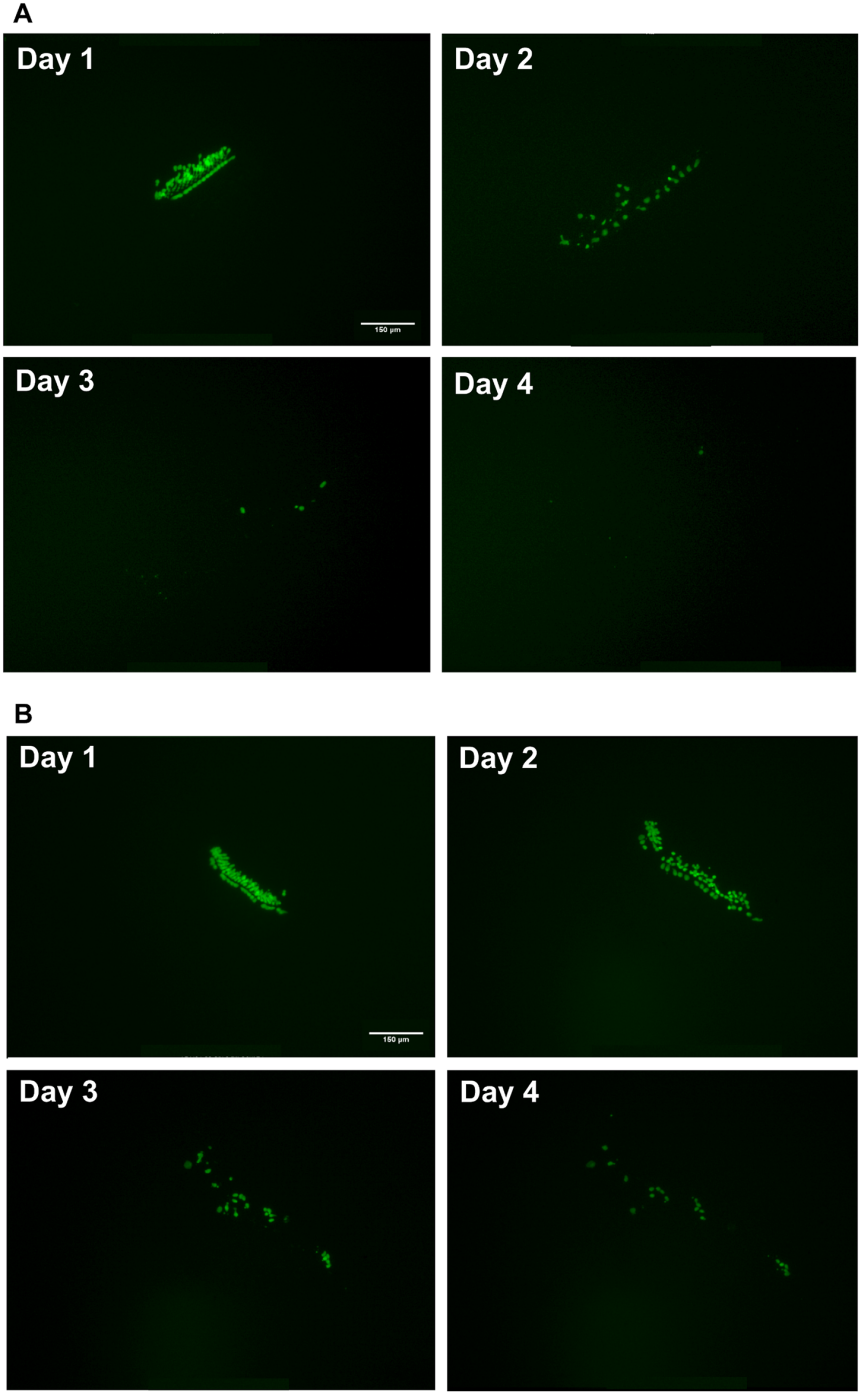
Positive control cultures. Sample montages of representative, positive control (gentamicin treated) oC micro-explants from the Library I (**3A**) and Library II (**3B**) screens. Gentamicin was added on D1, after 1 day of kinase inhibitor treatment.

Imaging of GFP-positive cells in negative and positive control wells showed a similar behavior of micro-explant cells. Negative control micro-explants showed high survival of GFP-positive HCs on D1-D2, with some loss apparent on D3 (Fig 2A and 2B), as is commonly seen in long-term neonatal oC cultures. Positive control micro-explants showed increasing losses of GFP-positive HCs from D1 onward (Fig 3A and 3B).

**Fig 4:**
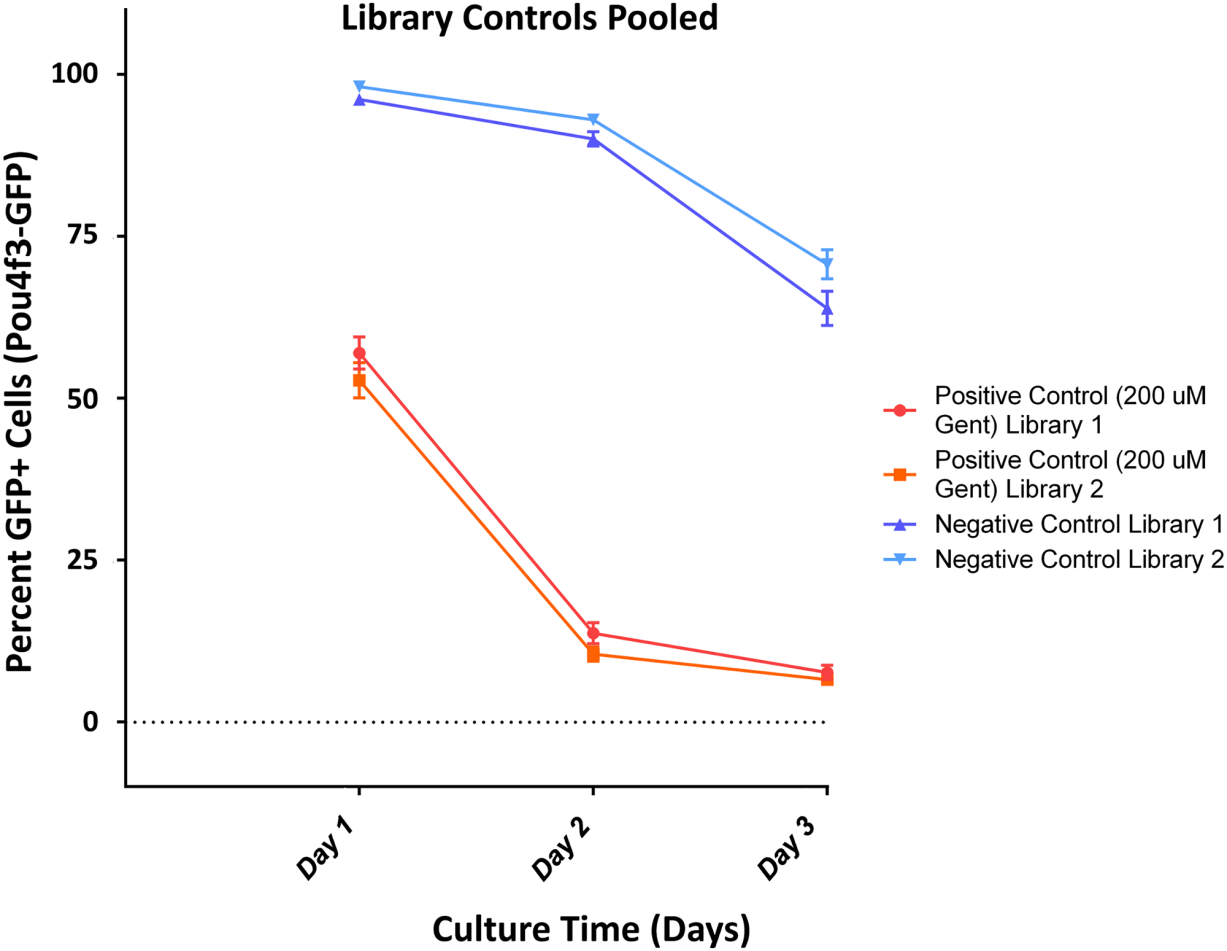
Quantitative analysis of positive and negative oC controls. Averaged cell counts from all control micro-explants from Library I and Library II plates, showing highly reproducible survival curves between D2-D4, referenced to the HC counts on D1. Error bars represent ±SEM.

HC counts from negative and positive controls were pooled for each library. They were converted to percent survival based on D0 HC numbers, prior to gentamicin exposure (Fig 4). Negative control micro-explants for Libraries I and II showed high levels of HC survival on both D1 (96% and 98%, respectively) and D2 (90% and 93%, respectively). By D3 negative control micro-explants showed reduced HC survival (64% and 71%, respectively). Positive control micro-explants from Libraries I and II showed significantly reduced survival on D1, after 24 hrs exposure to gentamicin (57% and 53%, respectively), and significantly lower survival after 48 hrs exposure on D2 (14% and 10%, respectively). D3 positive control micro-explants showed continued loss of GFP-positive cells (8% and 7% respectively). Repeated measures ANOVA followed with Fisher’s PLSD posthoc test showed a highly significant difference between negative and positive controls over time (p<0.0001), but no significant difference between the two negative control pools or the two positive control pools for Libraries I and II, despite the large number of samples (p = 0.1208 and p = 0.2659, respectively). Variability across cultures in the same condition and at the same time was low. The control micro-explants thus provided stable baselines against which the effects of inhibitor treatments could be evaluated.

### Immunohistological characterization of control oC micro-explants

**Fig 5:**
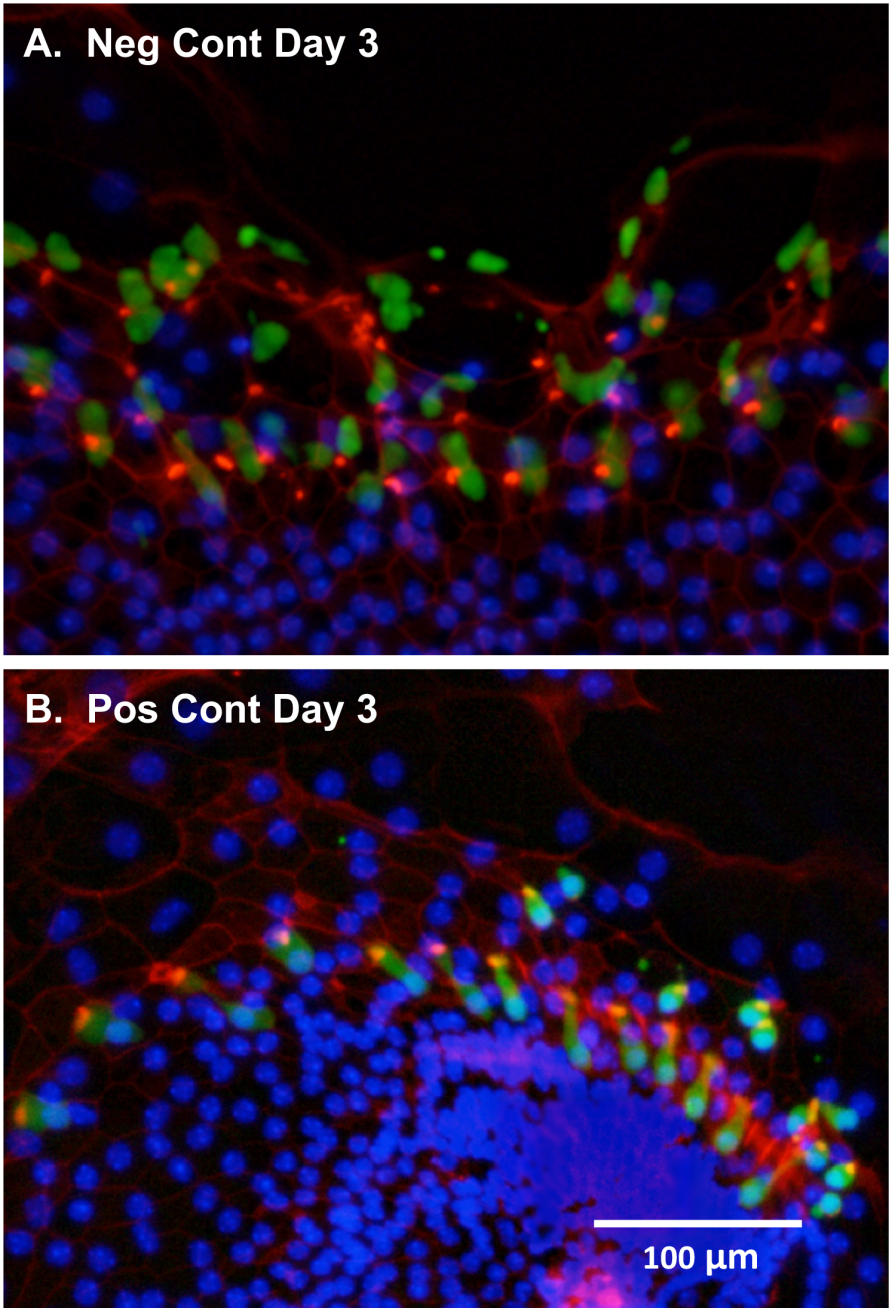
Morphology of positive and negative controls. Micro-explants of GFP-expressing HCs after 4 days in culture. The micro-explants have been stained with Texas-Red conjugated phalloidin to label the actin in HC stereocilia bundles and DAPI to label cell nuclei. The negative control is typical. The positive control micro-explant was chosen because a more than average number of HCs survived, so that HC morphology could be illustrated.

Negative control oC micro-explants were cultured for 4 days, representing the maximum number of days in the kinase inhibitor screen. After fixation and immunohiostochemistry (Fig 5A), counterstaining with DAPI revealed intact nuclei in all GFP-positive cells. Phalloidin staining revealed on the presence of stereocilia on almost all GPF-positive cells, confirming their identity as HCs and their general condition. Distortion of the epithelium, due to expansion and migration of nonsensory cells, was apparent, and is commonly seen in oC cultures over time [21]. Positive control micro-explants (treated with gentamicin alone) were also cultured for 4 days and immunostained in the same manner (Fig 5B). For the greatly reduced numbers of GFP-positive cells, DAPI-stained nuclei and phalloidin-positive stereocilia bundles remained present. As in the micro-explant illustrated, the great majority of outer HCs and many inner HCs were lost from gentamicin-treated micro-explants by D4.

### Results from libraries I and II

It should be noted that Libraries I and II terminology have no functional significance, but merely represent the plate division of the entire collection of compounds. The numerical nomenclature of compounds is preserved to permit ready reference to the manufacturer’s list of inhibitors and their properties listed on their website [22].

**Fig 6:**
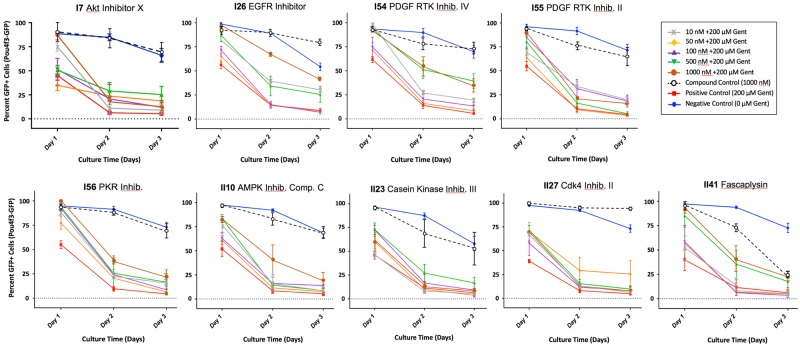
Protective kinase inhibitors. Survival curves for several inhibitors that exhibited significant (p < .05) protective effects after 1, 2 and/or 3 days of gentamicin exposure (D2-D4). For each inhibitor and dose, cell survival counts have been normalized to cell counts on D0, just before gentamicin was initially added, and pooled for all treated micro-explants.

**Fig 7:**
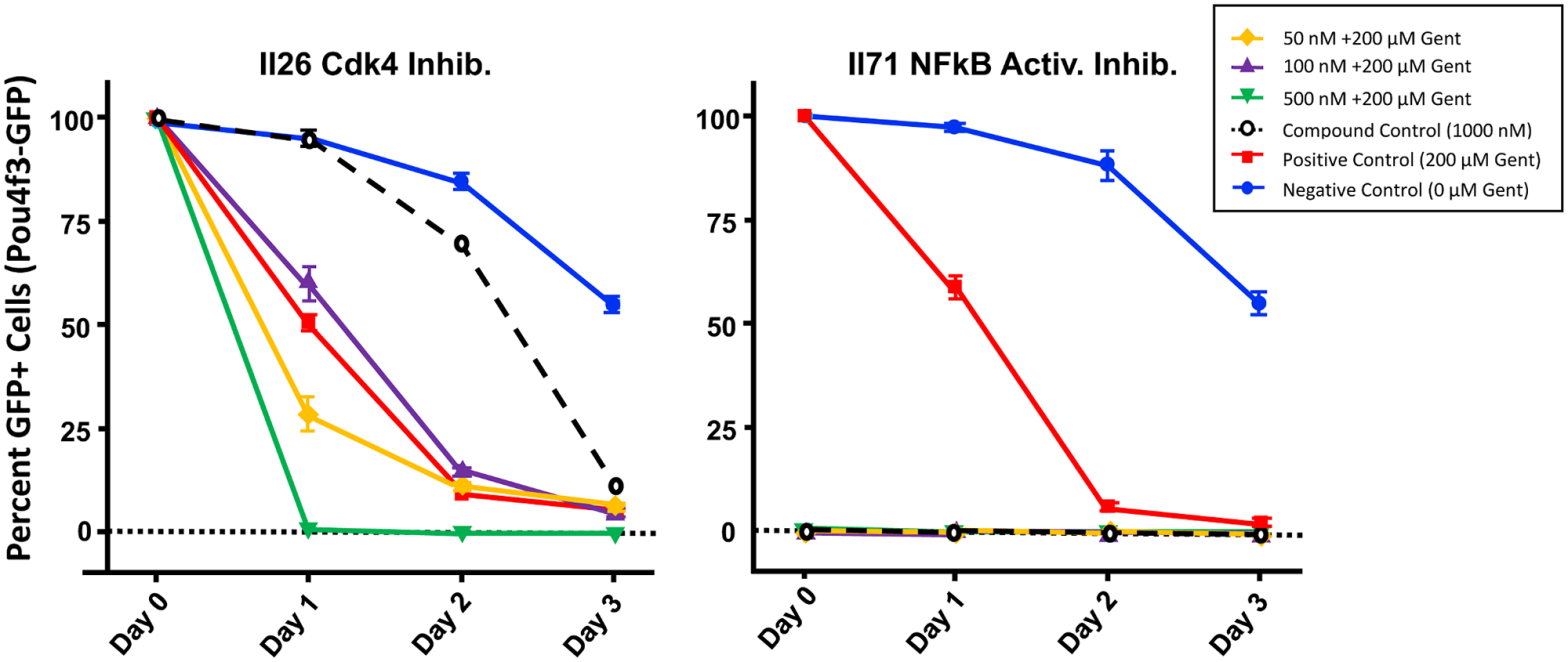
Damaging kinase inhibitors. Survival data are shown from D0, after 1 day of inhibitor treatment alone, and for days 1 to 3 of gentamicin treatment (D2-4), for two of the three inhibitors that significantly enhanced HC damage. Two modes of damage are illustrated. Inhibitor II26 had some toxic effect on HC survival on its own, but especially at 500 nM it strongly enhanced the damaging effects of the aminoglycoside. In contrast, 24 hrs of treatment with inhibitor II71 at all concentrations tested caused near total or complete loss of HCs on D1, prior to the addition of gentamicin, indicating very strong and independent toxicity.

Inhibitors found to influence GFP-positive cells in the micro-explant screen are summarized in Table 1. One-way ANOVA between inhibitor concentrations and controls followed by a Fisher’s PLSD posthoc test showed significant protection for 8 inhibitors in Library I, and 7 inhibitors for Library II. Examples of HC survival curves for several protective inhibitors are illustrated in Fig 6. In addition, 4 inhibitors were observed to have either a toxic effect alone in the micro-explant screen, prior to the addition of gentamicin, or to exert a synergistic damaging effect with gentamicin exposure. Examples are presented in Fig 7, and the results described in detail below.

**Table 1:** Kinase inhibitors influencing gentamicin-induced HC damage.

Library I Protective Inhibitors	Strength/Day of Protective Effect	Optimal Inhibitory Concentration	Target/IC_50_ information
**I5 Akt Inhibitor V Triciribine**	Weak D1 (100 nM p <0.01)	100 nM (D2)	IC_50_ = 20 nM HIV-1, HIV-2
**I7 Akt Inhibitor X**	Strong D1 (1000 nM, p <.01)	1000 nM (D1)	IC_50_ = 1000 nM Akt
**I26 EGFR Inhibitor**	Strong D1 (1000, 500 nM p <0.0001 Strong D2 (1000, 500 nM p <0.002) Strong D3 (1000, 500 nM p <0.004)	1000 nM (D1-D3)	IC_50_ = 63 nM EGFR
**I35 Flt-3 Inhibitor III**	Medium D1 (1000, 500 nM, p <0.04)	1000 nM (D1)	IC_50_ = 50 nM Flt3 260 nM cKit
**I54 PDGF Receptor TK Inhibitor IV**	Strong D1-D3 (500, 1000 nM p <0.0005)	500,1000 nM (D1-D3)	IC_50_ = 4.2 nM and 45 nM PDGFR-b and PDGFR-a, respectively; 22 nM, 100 nM,185 nM and 378 nM cAbl, Lck, c-Src, Fyn, respectively
**I55 PDGF RTK Inhibitor**	Strong D1 1000 nM p< .001; Medium D1 (100 nM p <0.01, Medium 500 nM (D2/3, p <0.02) Medium D2 (100 nM p <0.004) Medium D3 100 nM p <0.03)	1000 nM (D1)	IC_50_ = 4 nM PDGFR phosphorylation; 7.6 nM 500 PDGFR kinase activity; 234 nM c-kit receptor activity; 434 nM c-kit receptor phosphorylation
**I56 PKR Inhibitor**	Strong D1 (1000 nM p < .0001) Medium D1 (50 nM p <0.001, 100 nM p <0.0001, 500 nM p <0.0001)	1000 nM (D1)	IC_50_ = 210 nM for RNA- induced PKR auto- phosphorylation; 100 nM for rescue of PKR-dependent translation block
**I62 PP3**	Medium D3 (p = 0.008)	100 nM (D2)	IC_50_ = 2700 nM for EGFR
**II10 AMPK Inhibitor, Compound C**	Strong D1 (500, 1000 nM p <0.001) Medium D2 (1000 nM p <.01)	500 nM (D1	Ki = 109 nM in the presence of 5mM ATP and absence of AMP
**II23 Casein Kinase II Inhibitor III, TBCA**	Strong D1 (500, 1000 nM p <0.001, 100 nM p <0.01) Weak D2 (500 nM p <0.05)	500 nM (D1)	IC_50_ = 110 nM for CK2
**II27 Cdk4 Inhibitor II, NSC 625987**	Strong D1 (1000 nM 500 nM p <0.001, 100 nM p = 0.021, 50 nM p <0.001) Weak D2 (50 nM p <0.01) Weak D3 (50 nM p <0.02)	50 nM (D2-D4)	IC_50_ = 200 nM for Cdk4/D1
**II41 Fascaplysin, Synthetic**	Strong D1 (500, 1000 nM p <0.0001, Weak D1 (100 nM p <0.03, 50 nM p <.04) Medium D2 (500, 1000 nM p <0.001) Weak D3 (500, 1000 nM p <0.03)	1000 nM (D1-D3)	IC_50_ = 350 nM for Cdk4/D1
**II74 p38 MAP Kinase Inhibitor III**	Weak D2 (100 nM p = 0.02)	100 nM (D2)	IC_50_ = 380 nM for p38a
**II75 p38 MAP Kinase Inhibitor**	Strong D1 (100 nM p <0.02) Weak D2 (100 nM p <0.03, 500 nM p <0.03)	100 nM (D1-D2)	IC_50_ = 35 nM for p38MAPK
**II95 Tpl2 Kinase Inhibitor**	Medium D1 (50 nM p <0.03) Weak D2 (500 nM p <0.03) Weak D3 (500 nM p <0.03)	50 nM (D1)	IC_50_ = 50 nM for Tpl2 Kinase

### Protective library I inhibitors

Inhibitor 7 from Library I (I7) is an inhibitor of the Akt pathway, Akt Inhibitor X. A protective effect of I7 was observed throughout the period of culture at several concentrations.

I26 is an inhibitor of the epidermal growth factor receptor (EGFR). The protective effect of the inhibitor against 200 μM of gentamicin was strongest at the 10, 50 and 1000 nM concentrations, and occurred throughout the exposure period. The effect against HC death was strongest at D2 and D3 (post 24–48 hrs of culture with gentamicin); however, a protective effect was observed up to 72 hrs post exposure to gentamicin. Significant structural morphology differences were observed after culturing overnight with inhibitor alone, primarily that outer HCs appeared to move away from the row of inner HCs and form a bundle of cells.

I35, Flt-inhibitor 3, is an inhibitor of both Flt3 and c-kit at the concentrations used. It also has inhibitory effects on KDR, c-Abl, Cdk1, c-Src, and Tie-2; however, the inhibition of these molecules requires higher concentrations than those used in this assay. The protective effect was observed at 500 and 1000 nM concentrations, and was strongest at D1 after 24 hrs culture with gentamicin.

I54, PDGFR Inhibitor II, is an inhibitor with activity against PDGFR, c-Abl, Lck, c-Src, and Fyn at the concentrations used in this assay. A robust protective effect was observed at 500 nM concentration, and could be observed at D2-D4 (post 72 hrs of culture with gentamicin).

I55, PDGF RTK Inhibitor, is an inhibitor of PDGFR phosphorylation and c-kit. The protective effect was strongest at the 100, 500 and 1000 nM concentrations on D1 and D2, with some protective effect still observed on D3.

I56 is an inhibitor of RNA-dependent protein kinase (PKR) autophosphorylation. The protective effect of this inhibitor was observed at all inhibitor concentrations. It was maximal on D1, but continued through D3.

I62, PP3, was included in the library as a negative control for the Src inhibitor PP2. Its inhibitory effect was observed at D2 at the 100 nM concentration. I62 does have activity against EGFR and the EGFR inhibitor I26 did inhibit HC loss, but the IC50 of I62 against EGFR is 2.7 μM. It is also unclear why, at the 100 nM concentration and not the 500 nM concentration, I62 would have a protective effect and that it was seen only at D2. This may have been a false hit, and it is possible that additional replication with this inhibitor would yield no significant effect.

### Protective library II inhibitors

II10, compound C, is a selective inhibitor of bone morphogenetic protein (BMP) and is also an inhibitor of AMPK. The strongest protective effects were observed at 500 and 1000 nM concentrations on D1, with only 1000 nM being protective on D2 and loss of significant protection on D3.

II23, Casein kinase inhibitor 2, is an inhibitor of casein kinase 2 (CK2). The protective effect of II23 was observed at the 100 and 500 nM concentrations. The effect was strongest on D1, with a weak protective effect on D2 at the 500 nM concentration.

II27, Cdk4 inhibitor II, and II41, fascalypsin, are selective inhibitors of Cdk4. The strongest protective effect for II27 was observed on D1 at all concentrations. A protective effect was also observed in the 50 nM group on D2 and D3. Cdk4 inhibitor II alone enhanced HC survival above that seen with no treatment. The strongest protective effect of II41 was observed at the 500 and 1000 nM concentrations throughout the gentamicin exposure period. In contrast, treatment with 1000 uM fascaplysin alone proved to be toxic on D2 and D3.

II74, p38 MAPK Inhibitor III, II75, p38 MAPK Inhibitor and II7, PD169316, are selective inhibitors of the p38α MAPK. The strongest protective effect of II74 was observed at the 500 nM concentration on D2; of II75 was at the 100 and 500 nM concentrations on D1; and of II77 was at the 100 and 500 nM concentrations on D2. It should also be noted that there were additional p38 MAPK inhibitors in this library (II78, II83, II86, and II89) that did not exhibit a significant effect on HC survival.

Finally, II95, STO-609, is an inhibitor of Tpl2 kinase. The strongest protective effect was observed at the 50 nM concentration on D1 and D2.

### Inhibitors that increased HC damage

Library II, inhibitor 26: II26, another Cdk4 inhibitor, is suppresses the activity of both Cdk2 and Cdk4. It is more specific for Cdk4 (IC50 = 76 nM) than for Cdk2 (IC50 = 520 nM), and it also has activity against Cdk1 (IC50 = 2.1 μM), although this latter effect was likely not seen at the concentrations used in this assay. Interestingly, this inhibitor was only toxic at the 500 nM concentration and only in combination with gentamicin. The 100 nM and 50 nM concentrations had no significant effects on the normal course of the gentamicin toxicity (Fig 7, left panel).

Library II, inhibitor 71: II71, NF-kB activation inhibitor, suppresses NF-kB activation. The toxic effect of II71 was observed as a near total loss of HCs at all concentrations (50, 100 and 500 nM), even before the addition of gentamicin on D0. Not surprisingly, the 1000 nM compound alone was also highly toxic (Fig 7, right panel).

Library II, inhibitor 88: II88, SC-68376, is an inhibitor of Chk1 which also has inhibitor activities against Cdk1. It did not influence HC survival alone, but enhanced the toxicity of gentamicin. Enhancement was strongest at the 500 nM concentration on D1 after 24 hrs of gentamicin exposure, although significant increases in HC loss were also observed at the 100 nM and 50 nM concentrations by D2.

The general kinase inhibitor staurosporine, contained in both libraries (I95 and II92, respectively), also demonstrated a profound damaging effect on HCs when applied prior to gentamicin, comparable to II71 and similar to that reported in previous studies [23].

## Discussion

We developed an assay based on micro-explants of the neonatal mouse oC to screen kinase inhibitors that span the major families of the kinome for the ability to alter aminoglycoside damage to mammalian cochlear HCs. Positive hits were confirmed by re-screening. This resulted in the identification of contributions from diverse kinase families. Some of the kinases identified have been implicated previously in HC damage, while others are novel.

### Protective kinase inhibitors

I7 (Akt Inhibitor X). The protective effect of I7 was seen at the 500 and 1000 nM concentrations, and was strongest at D2 (after 48 hrs culture with gentamicin). There is prior evidence that Akt and PKC signaling is up-regulated following exposure to gentamicin in neonatal rat oC explants, and that inhibition of either pathway in conjunction with gentamicin exposure was found to worsen HC death in explants [24]. Why the results of I7 were opposed to these published findings is not clear. However, it is possible that I7 has a different influence on the three isoforms of Akt than the inhibitor used in the cited prior study. It is well known that different isoforms of signaling molecules can have distinct effects) [25], and differing roles of Akt isoforms in ototoxicity have been documented [26].

I26 (EGFR Inhibitor). EGFR has been shown to be expressed in rat oC at postnatal day 3 (P3) [27,28], and to be upregulated after aminoglycoside exposure rat oC explants [29]. However, treatment of explants with EGF did not show a protective effect [29]. Results with I26 suggest that one or more members of the EGF family may be involved in mediating HC damage. In other systems, EGFR inhibition has been shown to protect cells from ROS generation and cell death [30].

I35 (Flt-3 inhibitor 3). At the concentrations used in our study, I35 inhibits Flt3 and c-Kit. While Flt3 and c-Kit have not previously been implicated in HC damage, rare cases of deafness following c-Kit inhibition in patients has been reported [31,32]. This suggests Flt3 inhibition as a possible mechanism for the effects of I35. Flt3 inhibition has been shown to protect neurons against oxidative stress associated with glutamate toxicity [33].

I54 (PDGF Receptor TK Inhibitor IV). I54 is an inhibitor of not only PDGFR, but also c-Abl, Lck, c-Src, and Fyn at the concentrations used in this assay. A robust protective effect was observed at 500 nM concentration, and was present at D1-D3. c-Abl has been shown to be up-regulated in mouse oC following gentamicin exposure [34]. c-Src signaling has been implicated in noise-induced hearing loss, and treatment of chinchillas with Src inhibitors on the round window membrane prior to noise exposure was found to protect against HC death as measured by threshold shift and cyctocochleogram. c-Src signaling may mediate both mechanical and metabolic induction of apoptotic signaling in HCs, which means that it may also play a role in aminoglycoside-induced HC death due to aminoglycoside-induced dysregulation of cell metabolism. c-Src is involved both in apoptotic signaling due to cytoskeleton changes that stress cellular connections (anoikis) in the oC, and also in activation of reactive oxygen species (ROS) production [35]. A protective effect was observed via ABR threshold shift in noise-exposed chinchillas given a c-Src inhibitor plus n-acetyl cysteine (NAC), where NAC co-therapy provided no additional benefit [36]. As ROS generation is linked to aminoclycoside toxicity in HCs [7], prevention of ROS generation via blockage of c-Src in noise-induced hearing loss may overlap with gentamicin exposure in terms of protection. Furthermore, in a study of rats given cisplatin with or without a c-Src inhibitor, a protective effect was observed via both improved ABR threshold shift and OHC survival in the inhibitor treatment group [37]. The relatively strong effect of this inhibitor may be related to its broad inhibitory profile, which could have influenced more than one HC damage process.

I55 (PDGF Receptor TK Inhibitor). I55 inhibits phosphorylation of the PDGFR as well as of c-Kit. Its protective effect was strongest at the 500 and 1000 nM concentrations on D1 with a protective effect at 500 nM still observed on D2. Given the overlap in inhibition targets between this inhibitor and both I35 and I54, there may well be overlap in the mechanisms underlying the effects of these compounds.

I56 (PKR inhibitor). I56 inhibits RNA-dependent protein kinase (PKR) autophosphorylation. PKR-like endoplasmic reticulum kinase (PERK) is one mediator of unfolded protein ER-stress response via translation inhibitor eIF2α. ER stress has recently been implicated in aminoglycoside ototoxicity [38–40]. The eIF2α kinases share a large degree of homology in their kinase domains [41], so it may be reasonable to think that I56 has activity against PERK and/or have activity specifically against the pro-apoptotic UPR signaling mechanism. In mouse cardiomyocytes, cisplatin toxicity involves activation of PKR as measured via phospho-eIF2α [42]. The ototoxic effects of acetaminophen (APAP) have also been attributed to activation of ER stress via PERK activity increasing levels of phospho-eIF2α in HEI-OC1 cells [39].

I62 (PP3). I63 inhibits EGFR kinase. A protective effect was observed at D3 at 100 nM. I62 has activity against EGFR at an IC50 of 2.7 μM, so this is an unlikely effect for this compound. Thus, it is unclear why the 100 nM concentration and not the 500 nM would have a protective effect, or why this would only become apparent at D3. It is possible that additional replication with this inhibitor would reveal this to have been a false hit.

II10 (Compound C). II10 is a selective inhibitor of bone morphogenetic protein (BMP) as well as of AMPK. The strongest protective effect was observed at 500 nM concentration on D1, with only 1000 nM showing protection on D2. Because BMP seems unlikely to be involved in ototoxic signaling, our analysis of this assay focuses on the role of AMPK signaling in gentamicin-induced ototoxicity. It has been demonstrated that ROS activation of AMPK can lead to e2F1-mediated apoptotic signaling. This signaling pathway can be activated by hypermethylation of 12S rRNA by methyltransferase mtTFB1. In mtTFB1-overexpressing transgenic mice, progressive hearing loss is observed along with apoptosis in the stria vascularis and SGNs. The human mutation, A1555G in mtDNA, can cause maternally inherited deafness due in part to the same hypermethylation event leading to impaired mitochondrial ribosome function. Patient-derived A1555G cells with methylated 12S rRNA activate AMPK and trigger e2F1-mediated apoptosis *in vitro* [43]. Given the role of mitochondrial stress and ROS generation associated with gentamicin, blockade AMPK signaling to prevent e2F1-mediated apoptosis could be a plausible protective mechanism of II10. Conversely metformin has a protective effect in HEI-OC1 cells when they are treated with gentamicin via modulation of intracellular calcium flux and reduction of ROS species [44]. Metformin can activate AMPK signaling in both hepatocytes and skeletal muscle cells [45], so it is unclear if the activation of AMPK is responsible for the protective effect of metformin or if there are pleiotropic effects in oC cells beyond AMPK signaling. Furthermore AMPK has been shown to play a pivotal role in regulation of voltage-gated potassium (BK) channels, and in AMPK-knockout mice both BK channel levels and recovery from acoustic trauma were impaired compared to wild type despite the threshold shift being relatively similar [46]. Thus there may be several roles for AMPK signaling in the inner ear, and inhibition of those pathways having to do with pro-apoptotic signaling may cause a short-term protective effect against gentamicin.

II23 (Casein Kinase Inhibitor III). II23 is an inhibitor of casein kinase 2 (CK2). The protective effect of II23 was observed at 100 and 500 nM. II23 lost its protective effect at 1000 nM, perhaps due to the inhibition of other kinases at this dose. CK2 is a Ser/Thr specific kinase that is involved in a number of signal transduction pathways. There is some evidence to suggest that CK2 may play a role in apoptotic signaling, especially in response to oxidative stress. In a human neuronal blastoma cell lineage overexpressing α-synuclein, promotion of aggregates of α-synuclein due to oxidative stress treatment was linked to an increase CK2 and cathepsin D. Furthermore, CK2 inhibition was shown to lower α-synuclein in stressed cells, linking CK2 to the cellular stress pathway [47]. Another study showed that CK2 inhibition in rat PC12 adrenal cell line can lower the level of oxidative-stress induced apoptosis by suppressing a number of apoptotic pathways include caspase-8-, bid-, and mitochondrially-mediated apoptosis [48].

II27 (NSC 625987) and II41 (Fascaplysin). II27 and II41 are selective inhibitors of Cdk4/6, which are important for cell cycle G1 phase progression. In the avian inner ear, Cdk inhibitors like p27^Kip1^ have been shown to protect HCs from gentamicin-induced ototoxicity [49]. Thus it would appear that tightly regulating the cell cycle in HCs is important for their survival. The link between gentamicin and Cdk4 inhibition may be that cell survival is promoted due to a prolonging of cell cycle arrest by Cdk4 blockade during gentamicin exposure. While the HCs of the murine inner ear are post-mitotic by days 3–5 [50], it is possible that gentamicin damage forces HCs into the cell cycle pathway, as has been suggested by Tao and Segil [51]. The cell cycle can be fatal for post-mitotic cells [52]. Cell cycle arrest, induced by Cdk4 inhibition, could prevent or delay this process.

II74 (P38 MAPK Inhibitor III), II75 (p39 MPK Inhibitor) and II77 (PD169316). II74, II75 and II77 are inhibitors of the p38α MAPK. Their strongest protective effect was observed at the 500 nM concentration on D2. It has been shown in rat P3 oC culture that minocycline can decrease gentamicin-induced apoptosis by inhibiting phosphorylation of p38 MAPK, activation of caspase-3, and mitochondrial cytochrome c release; however, p38 MAPK inhibition alone was not as protective as the inhibition minocycline provided [53]. p38 MAPK phosphorylation has been demonstrated following noise exposure, and treatment with a p38 MAPK inhibitor reduced threshold shift and protected HCs in treated mice post-noise exposure [54]. The PKC inhibitor Gö6976 also has activity against p38 MAPK, and has been shown to protect neurons from lipopolysaccharide-induced inflammation and glial-mediated damage [55]. Interestingly, this same inhibitor, Gö6976, has been shown to be protective against gentamicin in the immortalized oC cell line OC-k3 [56]. Although PKCα activation was demonstrated in this paper, PKCα signaling was only present for the first 15 minutes of gentamicin exposure in these cells but the inhibitor treatment was effective up to 48 hrs post gentamicin exposure. It may thus be that p38 MAPK inhibition was the primary protective mediator in these cells. It should be noted that Gö6976 was an inhibitor (I31) in this screen; however, the concentration of gentamicin used in this assay was four times higher than in the assay where the protective effect was observed and thus may overshadow a protective effect for this compound. Similarly, several other p38 MAPK inhibitors in this assay (II78, II83, II86, and II89) were ineffective. This could be related to the strength of inhibition. Alternatively, it is possible that the inhibitors have differential effects on the four p38 MAPK isoforms [57]. In any case, the protective effective of three p38 inhibitors in our screen suggests that p38 MAPK signaling plays a significant role in play a role in gentamicin-induced ototoxicity, perhaps acting as a monitor for oxidative stress in HCs and initiating cell death.

II95 (Tpl2 Kinase Inhibitor). Tpl2 kinase is an inducer of several kinase pathways, including MAPK and IKK pathways. It has been shown to be critical in signaling for macrophage response to LPS via MEK and ERK1/2. There is evidence that Tpl2 kinase stability and expression is in turn regulated by NF-kB signaling [58]. There is no prior evidence that Tpl2 kinase has a role in aminoglycoside ototoxicity.

### Damaging kinase inhibitors

II26 (Cdk4 Inhibitor) is an inhibitor of both Cdk2 and Cdk4 at the concentrations employed. Taken alongside the protective effect of II41, a selective Cdk4/6 inhibitor, this result would appear to implicate Cdk2 as a protective regulator of HC survival. Cdk2 is critical for the transition from the G1 phase of the cell cycle to the S phase. This would implicate a prolonged G1 phase and subsequent delay of cell cycle progression as potentially damaging to HCs. This is in contradiction to the above interpretation regarding the protective effects of Cdk4 inhibitors. However, arguing against a role for Cdk2 in aminoglycoside-induced damage is the fact that three Cdk2 inhibitors in Library II (II30, II32, II33) showed no significant HC protection against gentamicin damage.

II71 (NF-κB Activation Inhibitor). A previous study of NF-κB inhibition reported a similar toxic effect on HCs *in vitro* [59], while NF-κB has also been shown to enhance HC death due to cisplatin [60]. In mice, there is also evidence that deletion of the p50 subunit of NF-κB can lead to enhanced noise-induced hearing loss as well as degeneration of SGNs although no decrease HC numbers was observed [24]. In contrast, Jiang et al. [61] found NF-κB to protect HCs from aminoglycoside ototoxicity. Our results support a role for NF-κB in promoting HC damage.

II88 (SB218078). II88 is an inhibitor of Chk1 and also has activity against Cdk1. Chk1 is a cell checkpoint regulator that is downstream of normal retinoblastoma protein (Rb) control in the inner ear and vestibular system. Rb is a cell cycle progression inhibitor, and deletion of Rb from the inner ear can cause irregular proliferation and subsequent apoptosis of cochlear HCs. Given that Chk1 has the ability to phosphorylate and inactivate Rb, it would be counterintuitive to assume that Chk1 inhibition would lead to increased toxicity [27]. It may therefore be useful to consider Cdk1 inhibition. The specificity of II88 to Cdk1 is around 250 nM, consistent with damage enhancement restricted to 500 nM. It would thus appear that the most toxic effects of II88 occur when Cdk1 should be inhibited effectively. Interestingly, like Cdk2, Cdk1 is required for progression of cells from G1 to S phase of the cell cycle. This supports the notion that this transition may somehow be protective for HCs. However, as with Cdk2, two more specific Cdk1 inhibitors in library II, II18 and II19, failed to influence HC survival.

The disparate effects of cell cycle inhibitors on gentamicin-induced cell death, with Cdk4 inhibitors protective and a Cdk2 inhibitor damaging, are difficult to reconcile. However, it has been noted that while Cdk4 inhibitors are highly effective in arresting cell cycle, inhibition of Cdk2 often does not reduce cell proliferation due to redundant cell cycle progression factors [62]. In any event, the disparity points out an advantage of a compound screen, in that the results do not fit neatly into current knowledge or theory.

### Advantages and disadvantages of the screening assay

The results of this study validate the use of neonatal organ of Corti micro-explants as a screening tool with which to evaluate the effects of pharmacological compounds on differentiated mammalian HCs. Screening assays have the advantage that they can test the effects of much larger numbers of compounds than more comprehensive studies. This allows the evaluation of potential therapeutic agents without *a priori* assumptions regarding the mechanisms of HC damage, and permits the discovery of potential HC protectants or damaging agents that might not have been predicted from current knowledge. In this screen, several novel HC protectants were uncovered. A screen also provides comparative information on the relative protective ability of compounds. Thus, we found that some compounds with similar kinase targets displayed quite disparate protective ability. A critical advantage is that the assay uses mammalian HCs rather than a cell line or HCs from a different vertebrate class. Only mammals possess the highly differentiated outer HC, which is the most sensitive to many forms of damage. Finally, the use of a transgenic in which HCs express GFP allows monitoring of HC loss throughout the assay, decreasing the number of samples required as well as experimental variability.

However, the nature of a screen means that the assay employed is necessarily constrained. In our case, as in most screens, an *in vitro* procedure is necessary in order to increase throughput. Dissecting and placing tissue in culture can of course alter the responses of cells. Because fully adult mammalian cochlear HCs do not survive in culture, we have used neonatal organ of Corti micro-explants. Neonatal HCs from rodents have significant differences from the inner ear sensory cells of adult humans. They are not only from a distinct mammalian species, separated by millions of years of evolution, they are also much more immature than any postnatal human HCs. That said, there are more similarities between murine developing and adult human HCs than there are differences. There have been many studies demonstrating that HCs from both species respond similarly to toxins [63], while genetic defects that influence mouse HCs often have similar effects on human HCs [64].

Another constraint is the limited number of conditions that can be evaluated, given that micro-explants cannot be generated in the much larger numbers possible with cell lines or zebrafish. We thus used only one rather high concentration of gentamicin. This potentially missed compounds that might have been protective at a lower aminoglycoside dose. While we used several concentrations of the screened compounds, dosages outside of this range might have proved more effective. Indeed, when additional dosages (10 and 1000 nM) were performed for positive hits, protection was often observed. Of course, if these additional dosages had been performed for all compounds it is possible that additional hits would have been discovered. However, this study was performed as a screen, and testing a larger number of dosages on all compounds would have made the screen less practical. That said, the results obtained with 1000 nM for the hits were generally positive, suggesting that we could have employed a wider range for the three dosages used in the initial screen.

Another limitation is the period of time over which even neonatal HCs can be maintained in culture. This necessitated that the pretreatment of micro-explants with compounds coincided with the period necessary for micro-explant attachment to the culture well. Free-floating micro-explants are difficult to reliably image, and thus HC counts were only be obtained after pre-treatment. This raised the possibility that compound toxicity during pre-treatment might be missed. While missing cells leave gaps in the regular array of inner and outer HCs, which provided one means by which to identify toxicity when the initial micro-explant images were obtained on D0, this is not an infallible measure. Also, the effects of compounds that might require a longer pretreatment period could have been missed.

These and other issues mean that compounds identified in the current screen need to be confirmed by more extensive studies and evaluated in adult animals *in vivo*, to identify those that might potentially be translatable to humans.

## Conclusions

This kinase screen has validated previous studies indicating the importance of several kinase pathways in HC damage due to ototoxic compounds. These include the p38 MAPK, cSrc, and G1 progression pathways. The screen has also identified novel potential mediators not previously shown to be involved in HC damage. These include Akt, EGFR, Flt3, c-Kit, AMPK, CK2 and Tpl2. These findings indicate the complexity of intracellular processes that can contribute to ototoxic HC damage. None of our inhibitors proved to provide 100% protection, also consistent with a damage process that involves multiple cellular pathways and components.

It is interesting to note that even when inhibitors of a particular kinase were effective in protecting HCs, other inhibitors of that same kinase were not. Since these inhibitors are all effective in other tissues and situations, this indicates that inhibitors have variable effects depending upon context. This may also reflect the diversity of kinases, many of which have different isoforms that potentially play different roles [23].

While a substantial number of kinase inhibitors were protective, none offered complete protection of HCs from damage. This is presumably related to the relatively high dose of gentamicin employed. However, it may also reflect the complexity of damage processes and signaling. This would be consistent with the results presented here and by many prior studies implicating multiple cellular processes that contribute not only to HC damage and but also to attempts by the HC to protect itself from injury. Inhibiting any one process may in the end be insufficient to provide complete protection in the face of a strong damaging stimulus. Supporting this idea is the finding that I54, an inhibitor with a broad range of targets, was one of the most protective inhibitors.

It is important to note that inhibitors of some kinases previously shown to be involved in HC damage were not effective in this screen. This includes especially inhibitors of the JNK and ERK MAPKs, which have been found to promote damage and survival, respectively, in aminoglycoside HC toxicity [65,66]. This may be related to the high dose of aminoglycoside used in our screen. Given the variability in results for other inhibitors targeting the same kinases, as noted above, this is perhaps not surprising.

## Supporting information

S1 Table Kinase inhibitor libraries I and II. (DOCX)
