# Supplementary material for: A kinase inhibitor library screen identifies novel enzymes involved in ototoxic damage to the murine organ of Corti
Source: PLoS One. 2017 Oct 19;12(10):e0186001. doi: 10.1371/journal.pone.0186001 (PMC5648133; doi:10.1371/journal.pone.0186001)
Supplement: S1 Table — (DOCX) [file pone.0186001.s001.docx]

**Supplementary Table 1.** **Kinase Inhibitor Libraries I and II.**

**Library I**

I1 DMSO

I2 AG1024

I3 AGL2043

I4 Akt Inhibitor IV

I5 Akt Inhibitor V, Triciribine

I6 Akt inhibitor VIII (Akt1/2)

I7 Akt Inhibitor X

I8 PDK/Akt/Flt Dual Pathway Inhibitor

I9 Aurora Kinase Inhibitor II

I10 Bcr-Abl Inhibitor

I11 Bisindolylmaleimide I

I12 Blank

I13 DMSO

I14 Bisindolylmaleimide IV

I15 BPIQ-I

I16 Chelerythrine Chloride

I17 Compound 56

I18 DNA-PK Inhibitor II

I19 DNA-PK Inhibitor III

I20 PI-103

I21 Diacylglycerol Kinase Inhibitor II

I22 Diacylglycerol Kinase Inhibitor II

I23 EGFR/ErbB-2 Inhibitor

I24 Blank

I25 DMSO

I26 EGFR Inhibitor

I27 EGFR/ErbB-2/ErbB-4 Inhibitor

I28 Flt-3 Inhibitor

I29 Flt-3 Inhibitor II

I30 cFMS Receptor TK Inhibitor

I31 Gö 6976

I32 Gö 6983

I33 GTP-14564

I34 Herbimycin A

I35 Flt-3 Inhibitor III

I36 Blank

I37 DMSO

I38 IGF-1R Inhibitor II

I39 IRAK-1/4 Inhibitor

I40 JAK Inhibitor I

I41 JAK3 Inhibitor II

I42 JAK3 Inhibitor II

I43 JAK3 Inhibitor II

I44 Lck Inhibitor

I45 LY 294002

I46 LY 303511

I47 LY 303511

I48 Blank

I49 DMSO

I50 PD 158780

I51 PD 174265

I52 PDGF Receptor TK Inhibitor II

I53 PDGF Receptor TK Inhibitor III

I54 PDGF Receptor TK Inhibitor IV

I55 PDGF RTK Inhibitor

I56 PKR Inhibitor

I57 PKR Inhibitor, Negative Control

I58 PI 3-Kg Inhibitor

I59 PI 3-Kb Inhibitor II

I60 Blank

I61 DMSO

I62 PP3 (EGFR Kinase inhibitor)

I63 PP1 Analog II, 1NM-PP1

I64 PKCbII/EGFR Inhibitor

!65 PKCb Inhibitor

I66 Rapamycin

I67 Rho Kinase Inhibitor III, Rockout

I68 Rho Kinase Inhibitor IV

I69 Staurosporine, N-benzoyl-

I70 Src Kinase Inhibitor I

I71 SU11652

I72 Blank

I73 DMSO

I74 Syk Inhibitor

I75 Syk Inhibitor II

I76 Syk Inhibitor II

I77 TGF-b RI Kinase Inhibitor

I78 TGF-b RI Inhibitor III

I79 AG 9

I80 AG 490

I81 AG 112

I82 AG 1295

I83 AG1296

I84 Blank

I85 DMSO

I86 AG 1478

I87 VEGF Receptor 2 Kinase Inhibitor I

I88 VEGF Receptor TK Inhibitor II

I89 VEGFR TK Inhibitor IV

I90 VEGFR2 Kinase Inhibitor II

I91 VEGFR2 Kinase Inhibitor III

I92 VEGFR2 Kinase Inhibitor IV

I93 DNA-PK Inhibitor V

I94 Aurora Kinase Inhibitor III

I95 Staurosporine, Streptomyces sp.

I96 Blank

**Library II**

II1 DMSO

II2 KN-62

II3 ATM Kinase Inhibitor

II4 ATM/ATR Kinase Inhibitor

II5 Alsterpaullone

II6 Alsterpaullone, 2-Cyanoethyl

II7 Aloisine A, RP107

II8 Aloisine, RP106

II9 Aminopurvalanol A

II10 AMPK Inhibitor, Compound C

II11 Aurora Kinase Inhibitor III

II12 Blank

II13 DMSO

II14 Aurora Kinase/Cdk Inhibitor

II15 Indirubin-3′-monoxime

II16 BAY 11-7082

II17 Bohemine

II18 Cdk1 Inhibitor

II19 Cdk1 Inhibitor, CGP74514A

II20 Cdk1/2 Inhibitor III

II21 Cdk1/5 Inhibitor

II22 Casein Kinase I Inhibitor, D4476

II23 Casein Kinase II Inhib. III, TBCA

II24 Blank

II25 DMSO

II26 Cdk4 Inhibitor

II27 Cdk4 Inhibitor II, NSC 625987

II28 Cdk4 Inhibitor III

II29 Cdc2-Like Kinase Inhib., TG003

II30 Chk2 Inhibitor II

II31 Compound 52

II32 Cdk2 Inhibitor III

II33 Cdk2 Inhibitor IV, NU6140

II34 Cdk/Crk Inhibitor

II35 ERK Inhibitor III

II36 Blank

II37 DMSO

II38 ROCK Inhibitor, Y-27632

II39 ERK Inhibitor II, FR180204

II40 ERK Inhibitor II, Neg. control

II41 Fascaplysin, Synthetic

II42 GSK-3b Inhibitor I

II43 GSK-3b Inhibitor II

II44 GSK-3b Inhibitor VIII

II45 GSK-3 Inhibitor IX

II46 GSK-3 Inhibitor X

II47 GSK-3b Inhibitor XI

II48 Blank

II49 DMSO

II50 SU6656

II51 GSK-3 Inhibitor XIII

II52 Isogranulatimide

II53 IC261

II54 IKK-2 Inhibitor IV

II55 Indirubin Derivative E804

II56 JNK Inhibitor II

II57 JNK Inhibitor, Negative Control

II58 JNK Inhibitor V

II59 JNK Inhibitor IX

II60 Blank

II61 DMSO

II62 MK2a Inhibitor

II63 JNK Inhibitor VIII

II64 K-252a, Nocardiopsis sp.

II65 Kenpaullone

II66 KN-93

II67 MEK Inhibitor I

II68 MEK Inhibitor II

II69 MEK1/2 Inhibitor

II70 MNK1 Inhibitor

II71 NF-kB Activation Inhibitor

II72 Blank

II73 DMSO

II74 p38 MAP Kinase Inhibitor III

II75 p38 MAP Kinase Inhibitor

II76 PD 98059

II77 PD 169316

II78 SB220025

II79 Purvalanol A

II80 GSK3b Inhibitor XII, TWS119

II81 H-89, Dihydrochloride

II82 SB 202474, Neg cont. p38 Inhib.

II83 SB 202190

II84 Blank

II85 DMSO

II86 SB 203580

II87 HA 1077, Dihydrochloride Fasudil

II88 SB 218078

II89 SC-68376

II90 SKF-86002

II91 Sphingosine Kinase Inhibitor

II92 Staurosporine

II93 STO-609

II94 SU9516

II95 Tpl2 Kinase Inhibitor

II96 Bl
